# Implications for agricultural sustainability: predicting the global distribution of *Ralstonia solanacearum* under current and future climate scenarios

**DOI:** 10.3389/fpls.2025.1548640

**Published:** 2025-08-04

**Authors:** Aya I. Tagyan, Omar Elghoul, Wael N. Hozzein, Walaa Rabie, Dalal Hussien M. Alkhalifah, Noura A. El-Far

**Affiliations:** ^1^ Department of Botany and Microbiology, Faculty of Science, Beni-Suef University, Beni-Suef, Egypt; ^2^ Applied Biotechnology Program, Faculty of Science, Ain Shams University, Cairo, Egypt; ^3^ Department of Plant Pathology, Plant Pathology Research Institute, Agricultural Research Center, Giza, Egypt; ^4^ Department of Biology, College of Science, Princess Nourah bint Abdulrahman University, Riyadh, Saudi Arabia; ^5^ Department of Microbiology, Faculty of Science, Ain Shams University, Cairo, Egypt

**Keywords:** *Ralstonia solanacearum*, bacterial wilt, climate change, species distribution modeling, maximum entropy modeling, agricultural sustainability, ecological impact, bioclimatic variables

## Abstract

**Introduction:**

A rapidly growing population and ongoing urbanization continue to strain agriculture’s capacity to maintain a stable food supply, both through direct impacts such as land reclamation and indirect effects driven by accelerating climate change. One of the major consequences of climate change is the shifting geographic range of infectious plant pathogens, particularly *Ralstonia solanacearum*, the causative agent of bacterial wilt. This pathogen poses a significant threat to several economically important crops including tomatoes, bananas, eggplants, and tobacco.

**Methods:**

To assess the current and future potential distribution of *R. solanacearum* under various climate scenarios, maximum entropy (MaxEnt) modeling was applied. This method was used to construct predictive maps based on environmental variables influencing the pathogen’s distribution.

**Results:**

The predictive models demonstrated high accuracy and performance, with an area under the curve (AUC) of 0.89 and a true skill statistic (TSS) of 0.94. Annual mean temperature was identified as the most significant environmental predictor. The present-day distribution map revealed an almost cosmopolitan range, while future climate change scenarios indicated substantial shifts in distribution across all continents.

**Discussion:**

These findings highlight the urgent need for implementing sustainable agricultural practices and developing novel, environmentally friendly methods to control the spread of *R. solanacearum*. This is especially critical in developing countries where agriculture is most vulnerable, to ensure food security under changing climate conditions.

## 1 Introduction

One of the most serious global challenges of the millennium is climate change. Climate change itself is defined as changes in the normal temperature and precipitation patterns (Dore, 2005), which are primarily caused by anthropogenic surges resulting from human population growth and industrialization (Mgbemene et al., 2016). The biggest contributors to climate change include the burning of fossil fuels (Wuebbles and Jain, 2001), changes in land usage (Dale, 1997), and solid waste landfills (Du et al., 2017). Many impacts brought upon by these activities are currently observable and also irreversible such as an increased likelihood of extreme weather events (Hashim and Hashim, 2016), increased incidence of natural disasters (Berlemann and Steinhardt, 2017), and ultimately ecological imbalances.

In fact, the Intergovernmental Panel on Climate Change (IPCC), a body of the United Nations responsible for advancing scientific knowledge on anthropogenic climate change, has warned in a report that the global mean temperature on the Earth may increase by up to 4 degrees Celsius by the end of the 21^st^ century as compared to preindustrial averages if vigorous mitigation efforts are not rapidly undertaken (Woodward et al., 2014).

All ecosystems are affected by climate change, as its impacts can range from posing a risk for extinction for organisms that are unable to adapt (Urban, 2015) while others may adapt by alternating behavior, morphology, or physiology (Chown et al., 2010), which may lead to range shifting (HilleRisLambers et al., 2013), which poses a risk for non-indigenous species becoming invasive (Moran and Alexander, 2014). These changes can have detrimental economic consequences (Batten, 2018).

Plants, the most economically important primary producers, and the foundation of virtually all food chains, are not exempt from the effects of climate change. Unfortunately, this means climate change represents yet an additional challenge to agriculture (Parry et al., 1990), which is already heavily strained by other factors such as plant diseases (Gilligan, 2007), rapidly growing populations (Pereira, 2017), and the subsequent urbanization leading to reclaiming agricultural land for non-agricultural purposes (Satterthwaite et al., 2010). It is of crucial importance to promote sustainable agricultural practices as well as limit the impacts of climate change to ensure a stable food supply. Climate change can also potentially present increased risks of plant diseases, given that pathogens and/or disease vectors can also be affected by range shifting (Moran and Alexander, 2014).

Agricultural plant diseases exhibit a wide range of classes and causes, and among the causes is a bacterium known *Ralstonia solanacearum*, which causes an infectious disease known as bacterial wilt (Prior and Fegan, 2005). *R. solanacearum* is a genetically diverse aerobic, nonsporulating, and gram-negative bacterium that can infect many economically important plants across a cosmopolitan geographical range; in fact, it has so many known strains and closely-related species that a species complex was developed for *R. solanacearum* (Genin and Denny, 2012).

*R. solanacearum* is a soil-borne bacterium (Wang et al., 2023) known to aggressively colonize the xylem from the roots upwards (Tans-Kersten et al., 2001) causing the plant to wilt. Among its potential host plants are potatoes, tomatoes, eggplants, peanuts, tobacco, bananas, and plantains, among others (Hong et al., 2012). Due to the large range of hosts, symptoms of infection can vary according to the host, but common characteristics include wilting and yellowing of younger leaves (García et al., 2019). In potatoes (*Solanum tuberosum*) specifically, *R. solanacearum* causes potato brown rot, which can cause losses of up to 75% of the agricultural yield (Messiha et al., 2007).

Due to the very large geographic and host range exhibited by *R. solanacearum*, calculating the exact economic loss caused by this bacterium is impractical, difficult, and likely to be inaccurate, but it is undoubtedly very large. There is also no standardized method of controlling or limiting the spread of bacterial wilt, although there are some attempts at developing biocontrol agents for this purpose (Ahmed et al., 2022). It is also worth noting that like its plant hosts and all other living organisms, *R. solanacearum* is also subject to the ecological impacts of climate change, which may alter the regions affected by bacterial wilt due to possible range shifting (HilleRisLambers et al., 2013).

The study of climate change and its impacts were revolutionized with the development of geographic information systems (GIS). A geographic information system is a computer system that can capture, store, retrieve, analyze, visualize, and/or otherwise manipulate geospatial data (Chang, 2017). It can be used to process data generated from remote sensing technology in a subsystem of GIS known as integrated GIS, which eases the compatibility and integration of different systems and increases the overall transparency between them (Hinton, 1996).

Driven by ever-improving computing performance, the integration of high-throughput GIS analysis techniques on high-throughput remote sensing data generated by increasingly advanced satellites has enabled the study many different characteristics of the Earth remotely, eliminating the need for field-based experiments that may be invasive and cost more, allowing the inference of the biophysical properties of species’ habitats (Kerr and Ostrovsky, 2003), monitoring land cover, its changes and their effects on living organisms (Foody, 2008), and meteorological and climatological studies (Samanta et al., 2012). Mathematical models of the climate known as general circulation models, for both the current scenario as well as possible climate change scenarios, have been developed using and/or integrated with geographic information systems, and many such models are freely available on the Internet (Weart, 2010).

The public availability of climate data in locations where a species is known to reside, as well as the rapid development of data science and machine learning algorithms, fueled the development of a technique known as species distribution modeling (SDM). Species distribution modeling algorithms depend on correlating the environmental characteristics of a species’ ecological niche with the occurrence data of said species in order to predict the probability of eco-suitability for this species across other geographical regions (Guisan and Zimmermann, 2000). These models have numerous ecological applications including but not limited to biogeography studies, conservation biology, climate change research (Guisan and Zimmermann, 2000), and invasive species management studies (Srivastava et al., 2019).

The advancement of these technologies facilitate the study of biological systems under pressure, such as modeling the interactions between a species and its environment (Franklin, 2010). In this study, we aim to employ species distribution modeling to assess the relationship between the climate and the global distribution of *R. solanacearum* in both the present time as well as its potential future distribution under different climate change scenarios in 2050 and 2070, to assess the bacterial wilt vulnerability of different geographical regions in the present as well as how it may change in the future.

## 2 Materials and methods

### 2.1 Occurrence data of *Ralstonia solanacearum*

**Figure 1:**
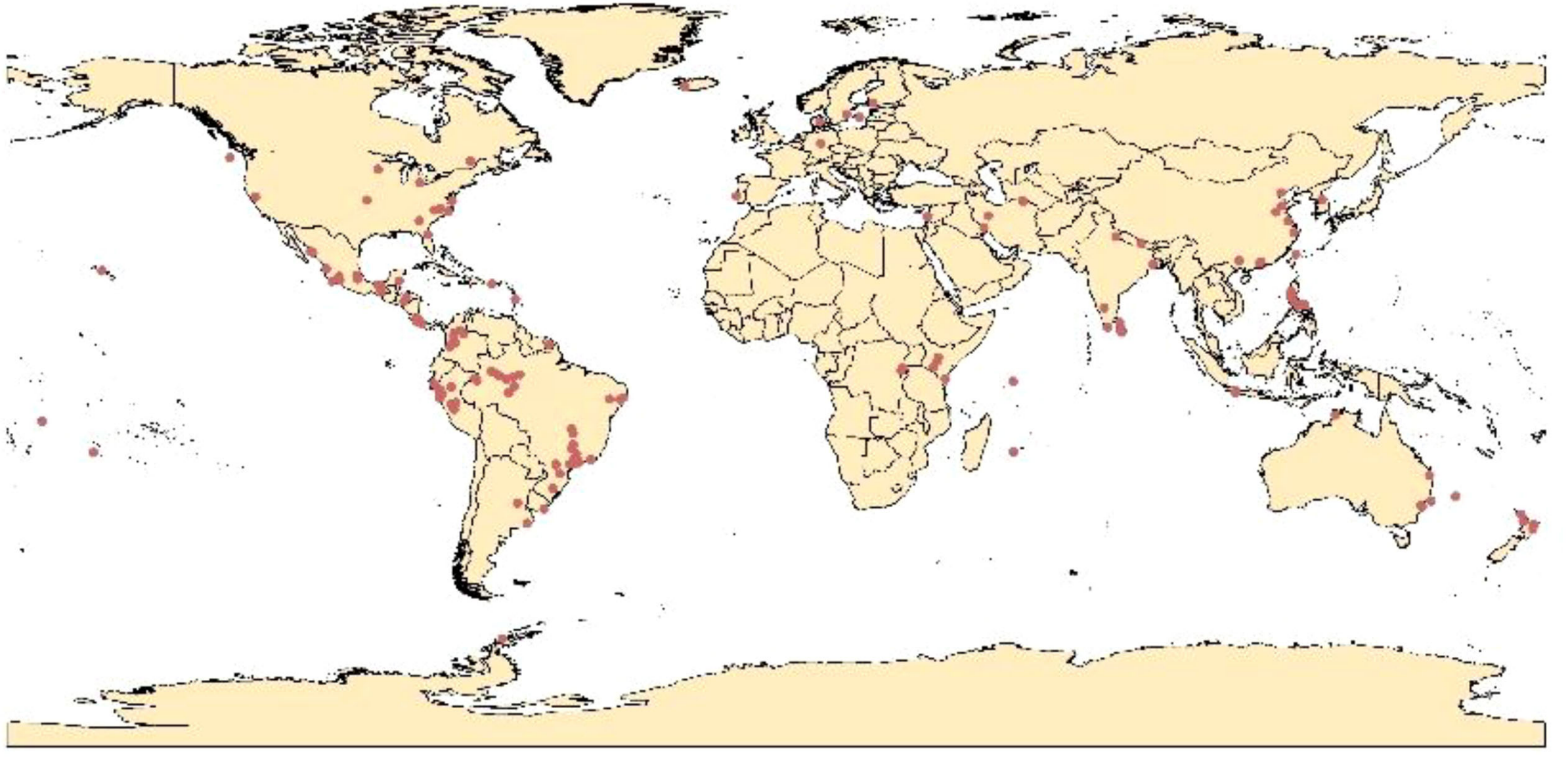
Occurrence records of *R. solanacearum* used to construct species distribution models.

The Global Biodiversity Information Facility (GBIF (accessed on 1 March 2023)) was used as a source of distribution points for *R. solanacearum* bacteria. The georeferenced occurrence points numbered 3982. Several preprocessing steps were conducted upon this data. First, the duplicate records (Hosni et al., 2020) and the records with high spatial uncertainty were eliminated from the study (Abou-Shaara et al., 2021). Furthermore, spatial rarefication of the data via ArcGIS was implemented to prevent any spatial redundancy and minimize sampling bias (Hosni et al., 2020; Abou-Shaara et al., 2021). The remaining 384 records were saved in the comma-delimited values (CSV) format and used to predict the global current and future distribution of *R. solanacearum* (**Figure 1**).

### 2.2 Environmental covariates

Bioclimatic data with a spatial resolution of roughly 5 km^2^ was retrieved from (www.worldclim.org). The current global climate was represented by a total of 19 climatic variables, which were originally calculated using monthly temperature and rainfall measurements gathered from meteorological stations between 1950 and 2000. These layers were converted to ASCII format with ArcGIS v.10.7 and used to assess the most crucial biological factors that contribute to the present model of habitat suitability for *R. solanacearum*. Due to spatial aberrations in those variables, bioclimatic variables 8–9 and 18–19 were removed from the study (Hosni et al., 2022b). These layers are known to have spatial anomalies between adjoining pixels due to their combining temperature and precipitation data, which causes discontinuities in their interpolated surfaces, particularly near the equator (2024). To reduce the multicollinearity among the remaining 15 bioclimatic variables, Pearson correlation was used, where highly correlated variables (|r| ≥ 0.8) were removed from the study (Hosni et al., 2022a; Hosni et al., 2022c). Ultimately, only five bioclimatic layers were used for further analysis. To account for the distribution of *R. solanacearum* based on carbon dioxide emission in the future, we used parallel datasets for the global climate model (GCM) from two representative concentration pathways (RCPs) 2.6 and 8.5 for 2050 (average of predictions for 2041 2060) and 2070 (average of predictions for 2061-2080) (Hijmans et al., 2005). These bioclimatic covariates were also converted into ASCII format via ArcGIS v.10.7. The climatic data for the chosen variables was then extrapolated using the Meteorological Research Institute’s (MRI-CGCM3) global climate model to the years 2050 and 2070 in order to determine how climate change may affect the suitability of *R. solanacearum* habitat in the future. This information is included in the most recent GCM climate estimates used in the IPCC Fifth Assessment Report.

### 2.3 Ecological niche modeling approach

Maxent v3.4.3e’s maximum entropy approach was used to calculate the ecological niche and habitat appropriateness of *R. solanacearum*. This approach is characterized by having the ability to use presence-only records to produce models (Byeon et al., 2018). Moreover, it can eliminate duplicate data in the same cell and is useful in modelling studies with limited sample sizes (Elith et al., 2006; Phillips et al., 2006). For these models, 25% of the records were utilized to test the model while 75% of the records were used for training (Kessler et al., 2019; Jiufeng et al., 2020). The maximum number of background points and iterations allowed were 10,000 and 1000, respectively (Abou-Shaara et al., 2021). Furthermore, a 10-fold cross-validation was performed, which enhanced the model’s performance (Kessler et al., 2019; Hosni et al., 2022b).

### 2.4 Model evaluation

The area under curve (AUC) is used to test the performance of the resulted models (Hosni et al., 2020). The range of the AUC is 0.5 to 1.0, and values above 0.9 are considered to indicate excellent model performance (Mulieri and Patitucci, 2019). Key bioclimatic factors were discovered using the jackknife test to evaluate the probable spread of the target species (Mulieri and Patitucci, 2019). Furthermore, the true skill statistic (TSS) was used to quantify the accuracy of the model (Allouche et al., 2006). The TSS value can range from -1 to 1, with positive values close to 1 suggesting a high relationship and negative values signifying a weak association between the distribution and the predictive model (Allouche et al., 2006; Hosni et al., 2022a).

### 2.5 Model visualization

The models outputted from Maxent were visualized as a world map and exported as an image using ESRI ArcMap v10.7. They were classified into different categories according to the range of probability of suitability using Jenks natural breaks optimization and then color-coded to ease visual interpretation of the models.

The models were then classified into a presence-absence map using a threshold of 0.6 probability of suitability where values greater than or equal to this threshold were considered present (represented by ones), and below absent (represented by zeroes). This threshold was chosen as it captures the area where approximately 90% of the training records are present so that the models can account for potential error margins. To ease interpretation of the future projected distribution maps, calibration models were calculated by subtracting the classified values of the current distribution from those of each of the future distribution models (Zhang et al., 2019a). The final map would then contain values ranging from -1 to 1, where positive values indicate gained range, negative values indicate lost range, and zeroes represented unchanged range. Each of the aforementioned classes was then color-coded and exported as an image as well.

## 3 Results

### 3.1 Model performance and contribution of bioclimatic covariates

**Figure 2:**
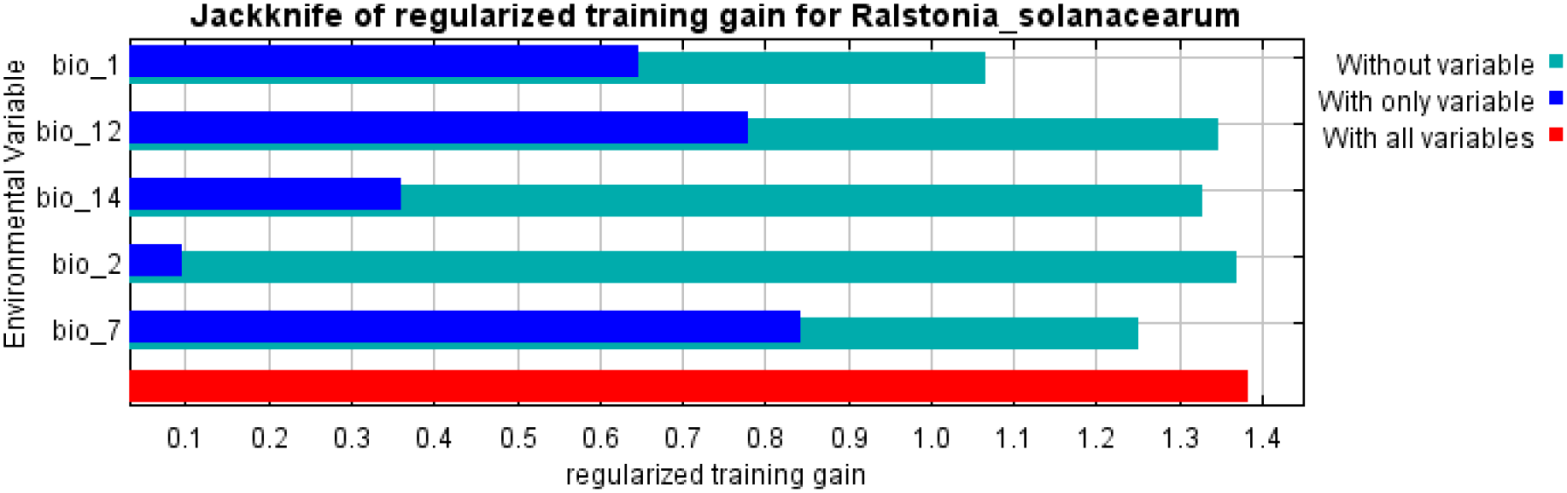
Jackknife test of the significant environmental covariates for *R. solanacearum*.

The predictive Maxent model showed a high AUC value equal to 0.89 (± 0.001). Generally, continuous species distribution modeling (SDM) showed more AUC values than discontinuous SDM. Furthermore, the TSS value are very high equal to 0.94 which indicate excellent model performance. Usually, TSS values of more than 0.5 are acceptable. The jackknife test of the predictive model illustrated the percentage contribution of the bioclimatic variables (**Figure 2**; **Table 1**).

**Table 1:** Percent contribution of the significant environmental covariates for *R. solanacearum*.

Bioclimatic variable	Description	Percent contribution
bio_1	Annual mean temperature	36.4%
bio_7	Temperature Annual Range	28.8%
bio_12	Annual Precipitation	22.7%
bio_14	Precipitation of Driest Mont	10.8%
bio_2	Mean Diurnal Range (Mean of monthly max temp—min temp)	1.2%

The annual mean temperature (bio_1) was the most effective bioclimatic covariate by percentage contribution followed by Temperature Annual Range (bio_7), Annual Precipitation (bio_12), Precipitation of Driest Month (bio_14), and Mean Diurnal Range (bio_2) respectively. Besides, the response curves of (bio_1) as the most important environmental factor ranged from 10°C to 30°C (**Supplementary Figure S1**).

### 3.2 Current habitat suitability model for *Ralstonia solanacearum*

**Figure 3:**
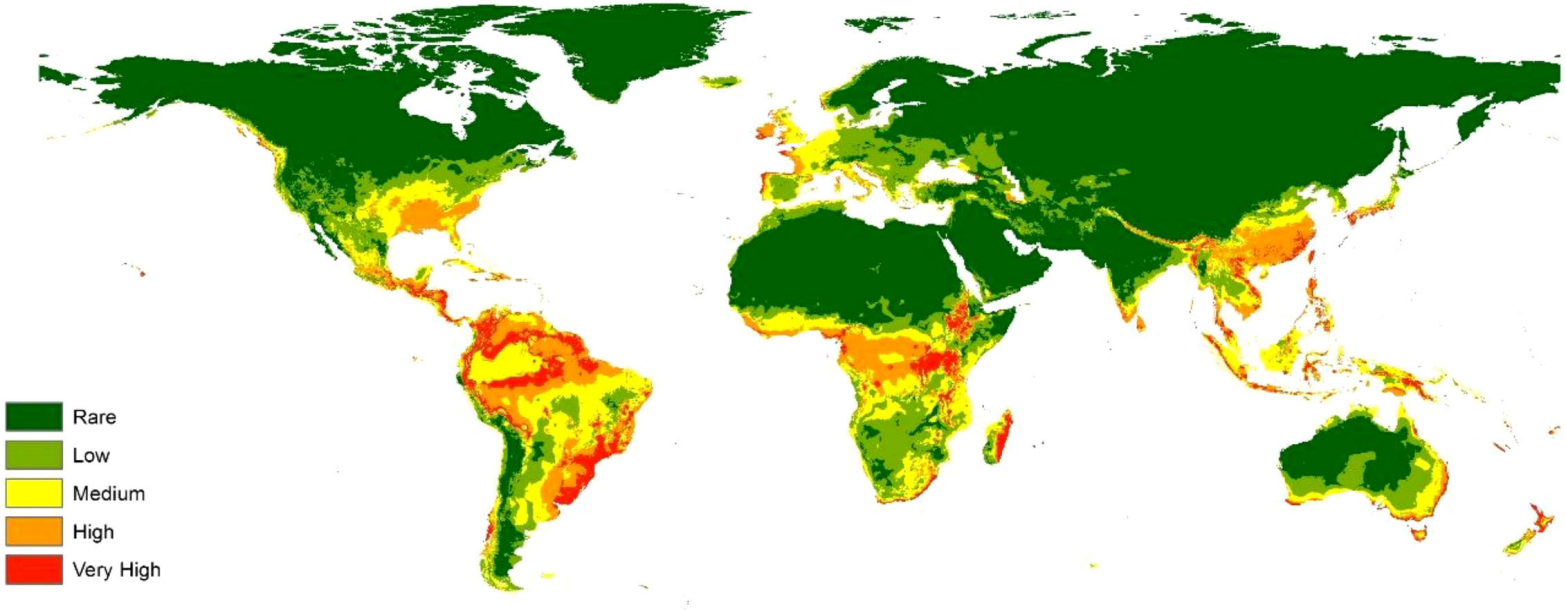
Global distribution map of *R. solanacearum* in the current climate scenario.

The current predictive map represented the bacterial actual situation with a global dispersion (**Figure 3**).

**Figure 4:**
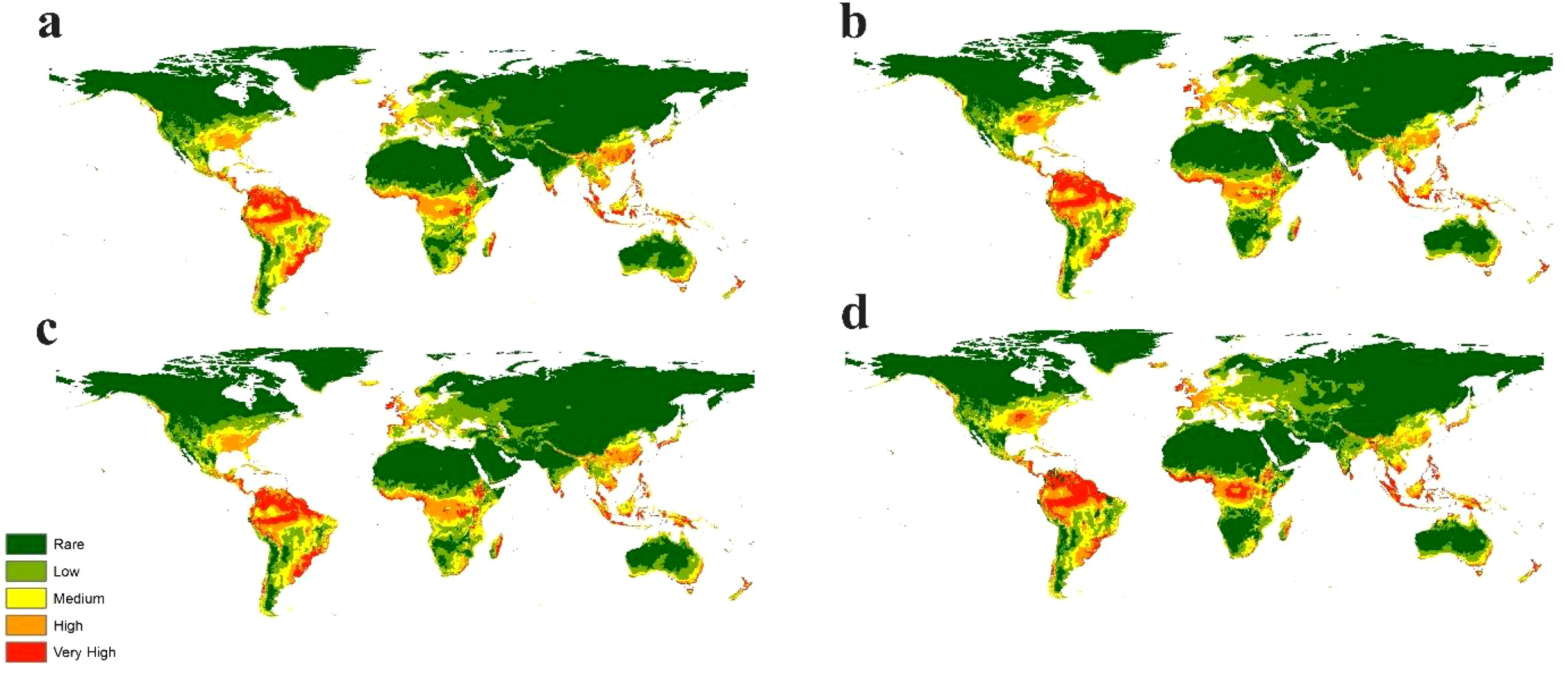
Global distribution map of *R. solanacearum* in the future climate scenarios under **(a)** RCP 2.6 in 2050 **(b)** RCP 2.6 in 2070 **(c)** RCP 8.5 in 2050 and **(d)** RCP 8.5 in 2070.

In Europe, the southern coast including Italy and Greece showed medium and high suitability for *R. solanacearum*. Also, western parts of France, Germany, UK, Ireland, and many parts in Portugal displayed very high suitable conditions for dispersal. The other territories of the old continent illustrated low suitability (**Figure 4**). In Africa, Equatorial and subequatorial belt showed high and very high eco-suitability including Ethiopia, Uganda, Kenia, Democratic Republic of Congo, Cameron till Ivory Coast and Sierra Leone. Besides, Eastern parts of South Africa and Madagascar showed very high suitability (**Figure 4**). In Asia, South India, Sri Lanka, approximately all Chinese territories, Philippines, Indonesia, Papua New Guinea, showed high and very high suitability. The Americas showed high and very high suitability in different parts including southern-east US, Mexico, Honduras, Nicaragua, and all Latin America excluding northern Chile and South Argentina (**Figure 4**). Finally, Eastern coast of Australia and many parts of New Zealand exhibited very high suitability for *R. solanacearum* (**Figure 4**).

### 3.3 Future habitat suitability model for *Ralstonia solanacearum*

The predictive Maxent models for future global distribution of *R. solanacearum* under the future climatic change scenarios of RCP 2.6 and RCP 8.5 for 2050 and 2070 is shown in **Figure 4**.

**Figure 5:**
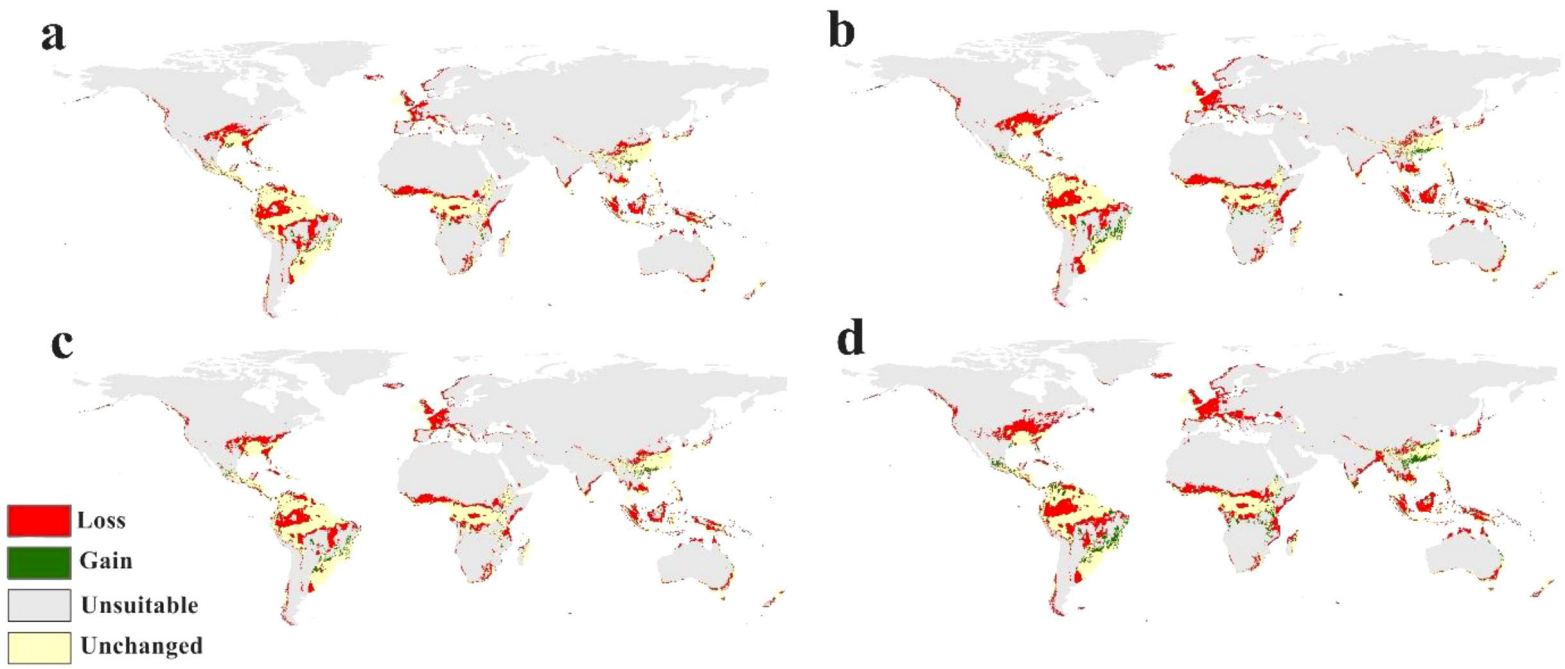
Global distribution calibration map of *R. solanacearum* in the future climate scenarios under **(a)** RCP 2.6 in 2050 **(b)** RCP 2.6 in 2070 **(c)** RCP 8.5 in 2050 and **(d)** RCP 8.5 in 2070.

In Europe, our predictive models displayed changing in suitability lower degrees in different parts including UK, Germany, Portugal, and Italy, while other parts showed increasing in suitability including Turkey and Bulgaria (**Figures 4**, **5**).

In Africa, great loss of habitat in many parts illustrated from Ethiopia in the easy to Sierra Leone in the west (**Figures 4** and **5**). In Asia, Northern parts of China showed loss of habitat while the eastern parts great gain. Also Southern Indian continent, South Indonesia and Malaysia displayed Loss in fitness in the future models (**Figures 4**, **5**). Furthermore, the future scenarios in the Americas predicted Loss in habitat in southern-east US, Mexico and increasing habitat suitability in Brazil and Peru from Latin America (**Figures 4**, **5**). Australia and New Zealand showed significant loss in habitat (**Figures 4**, **5**).

## 4 Discussion

Bacterial wilt is an infectious bacterial plant disease caused by *R. solanacearum* that represents a serious challenge to agriculture worldwide, with notorious consequences to the food supply (Wang et al., 2023). *R. solanacearum* is known to aggressively colonize the xylem vessels of its host plant despite the low nutritional content of the xylem sap (Tans-Kersten et al., 2001). This study aimed to assess the relationship between climate and the global distribution of *R. solanacearum*, which may serve to predict areas with more vulnerability to bacterial wilt that may benefit by avoiding planting crops that can be hosts to this bacterium, as well as predict the future distribution of *R. solanacearum* under different climate change scenarios to allow for communities to adequately prepare for possible ecological changes.

To this end, maximum entropy modeling was employed (Phillips et al., 2006) to construct species distribution models for *R. solanacearum* in the current climate scenario to predict its geographical distribution in the present, as well as in different future climate change scenarios to predict inferred infection incidence in the near future. The performance of the models were evaluated according to their mean AUC and TSS values and were deemed to be of excellent accuracy (Phillips and Dudík, 2008). The environmental covariates that affected the geospatial distribution of *R. solanacearum* to the greatest extent were also shown to be bio_1 (annual mean temperature), bio_7 (temperature annual range), bio_12 (annual precipitation), bio_14 (precipitation of driest month), and bio_2 (mean diurnal range) in descending order of significance. Interpretation of the response curves (**Figure 3**) also clarifies the almost cosmopolitan range of *R. solanacearum*, as it was shown to find annual mean temperatures suitable in a very wide range, as much as from 10°C to 30°C. The models were then visualized using ESRI ArcMap 10.3 (Zhang et al., 2019b).

The current potential distribution of *R. solanacearum* (**Figure 4**) shows medium-to-high environmental suitability for *R. solanacearum* in most of south and west Europe, most of Sub-Saharan Africa, Madagascar, South India, Sri Lanka, China, the Philippines, Indonesia, and Papua New Guinea. The Americas also showed high to very high suitability across most of its ranges, as did the eastern coast of Australia and most of New Zealand. This represents a potentially drastic threat to agriculture in the aforementioned regions and territories. For example, South America is well known for its banana production, which is devastated by the presence of multiple *R. solanacearum* strains that can cause a variety of bacterial wilts of banana (Fegan and Prior, 2006). Likewise, potato production in India is also severely impeded by potato brown rot caused by *R. solanacearum* infection from up to 75 different strains of the bacterium (Sagar et al., 2014). Tobacco production is also impeded by the presence of a wide variety of diverse *R. solanacearum* strains in China (Liu et al., 2017).

In the aforementioned regions and territories, there exist a variety of strategies that attempt to control bacterial wilt. One such approach is crop rotation, which is depends on alternating planting host and non-host plants. In one study, a one-season crop rotation strategy involving tomato-maize-tomato, bacterial wilt incidence was reduced by between 6% and 16%, while a two-season crop rotation strategy involving tomato-maize-common bean-tomato resulted in a 29% reduction in wilts (Ayana and Fininsa, 2017). In another study, rotating potato-lablab-potato resulted in a 19.9% increase of potato yield as compared to the yield when impacted by *R. solanacearum* (Mwaniki et al., 2017).

Several studies have shown that coinfection with root-knot nematodes, such as *Meloidogyne javanica*, can further intensify disease severity in chili, further complicating management efforts (Asghar et al., 2020). However, recent work has demonstrated that the use of *Trichoderma harzianum* as a biological control agent can significantly reduce wilt severity in tomatoes under dual infection by *R. solanacearum* and *Meloidogyne incognita* (2024). This highlights the promise of biocontrol as a novel and environmentally clean alternative to traditional chemical-based strategies. As conventional pesticide methods face increasing scrutiny for their ecological impacts and declining effectiveness, biocontrol agents such as *T. harzianum* offer a sustainable path forward in managing agricultural pests and complex pathogen interactions (Yaseen et al., 2024). This is especially relevant in the context of climate change, which is expected to intensify pathogen pressure and alter disease dynamics (Raza and Bebber, 2022).

Another approach to control *R. solanacearum* is to promote the breeding of resistant plants. This has several obstacles as consideration needs to be taken to combine both resistance with desirable agronomic traits, as well as consideration for resistant plants that may host *R. solanacearum* without displaying any infection symptoms. Additionally, the genetic components of plant resistance were shown to be polygenic in tomatoes, tobacco, and eggplants, and thus the development of resistant crops is impeded by the difficulty of genetically engineering a large number of genes that may also be linked to agronomically undesirable traits (Huet, 2014).

A more novel strategy to control *R. solanacearum* is to use copper oxide nanoparticles (CuO NPs) loaded with streptomycin, which was shown in one study to increase the protection of potatoes by 55.8% and reducing the disease severity by 37.5% in the infected potato plants (Attia et al., 2022). However, this approach is also impeded by the fact that some strains of *R. solanacearum* exhibit resistance to copper, as well as possible unintentional promotion of antibiotic resistance to the used antibiotics (Wang et al., 2013).

More novel methods to control *R. solanacearum* are currently being studied. In one study, pretreatment of tomato seedlings with a lytic phage drastically lowered the penetration, growth, and movement of *R. solanacearum* cells inoculated on the roots of the plants, and the plants showed no symptoms of wilting whereas untreated plants wilted 18 days post-inoculation with the bacteria (Fujiwara et al., 2011). Other biocontrol agents that show promising results for use against *R. solanacearum* include *Bacillus megaterium*, *Enterobacter cloacae*, *Picbia guillermondii*, and *Candida etbanolica*, where they all showed high potential for bacterial wilt suppression as well as increased fruit weight, plant biomass, and plant height (Nguyen and Ranamukhaarachchi, 2010). More studies are needed to further evaluate the safety and practicality of this approach.

As for the models illustrating the predictions for the different possible climate change scenarios (**Figures 5**), the calibration maps show substantial global range shifts in the distribution of *R. solanacearum* that intuitively increase in severity with increasingly severe possible climate change scenarios. The maps illustrate a net loss of range throughout most of West Europe as well as most of Sub-Saharan Africa. Interestingly, there is also a significant range gain in Brazil, Peru, and eastern regions of China. A significant proportion of the range of *R. solanacearum* is also predicted to remain unchanged in the future climate change scenarios used in this study.

Unfortunately, this means there is a growing threat in the regions and territories that exhibit either unchanged suitability or increasingly suitable environments for survival of *R. solanacearum* in a future environment that will also be more severely impacted by climate change. Even for the geographical ranges that will exhibit a net loss in suitability for *R. solanacearum*, it is worth noting that agriculture will likely still be increasingly challenged by the other impacts of climate change, that are not limited to bacterial wilt alone.

This study highlights that the ecological effects of climate change go far and beyond wildlife extinctions (Urban, 2015). The changes brought upon by climate change can directly affect our ability to provide a stable food supply in a world where agriculture is already strained by increasing populations and land reclaiming for urbanization and industrialization purposes.

However, a key limitation of this modeling approach is the reliance on presence-only records without corresponding absence data. Although MaxEnt is known to perform well under such conditions, it estimates relative suitability rather than absolute occurrence probabilities. This can lead to a potential risk of overprediction, particular in under-sampled regions (Phillips et al., 2006). The lack of absence data also limits the ability of the model to distinguish between unsuitable and unreported areas, introducing sampling bias if sampling effort is spatially uneven (Yackulic et al., 2013). The latter risk was addressed through spatial filtering of the occurrence data but should still be acknowledged. Incorporating absence data may improve the distribution accuracy of these models.

Although our models proficiently delineate the climatic determinants of *R. solanacearum* dispersion, we recognize that soil characteristics (e.g., pH, moisture, texture), elevation, and land-use practices are equally vital for bacterial viability and dissemination. These elements affect host accessibility, pathogen survival, and disease progression. Our research emphasizes climate variables because of their significant influence on broad-scale habitat suitability in both present and future scenarios, together with the global consistency and accessibility of climate forecasts relative to the spatially heterogeneous nature of soil or land-use data that are also not available for the future on a global scale. Future research may use these localized features to enhance predictions, especially for regional management techniques. Our climate-focused method establishes a solid basis for recognizing macro-scale changes in susceptibility to bacterial wilt due to climate change.

Finally, it is also worth noting that in this study, species distribution models for *R. solanacearum* were constructed using only bioclimatic variables alone, which provide a satisfactory estimation of its suitable geographic ranges, but that this estimation may be further improved via the use of more environmental covariates. For instance, a past study reported on migration of *R. solanacearum* from the Chinese lowlands to the highlands (Liu et al., 2017), suggesting that an additional possible significant environmental predictor may be elevation (Booth, 2022). Given that the *R. solanacearum* is also a soil-borne bacterium, incorporating additional environmental covariates such as soil type, soil composition, and soil chemical properties may also serve to improve the accuracy of the predictions.

## Data Availability

The original contributions presented in the study are included in the article/**Supplementary Material**. Further inquiries can be directed to the corresponding author/s.
