# Supplementary material for: Implications for agricultural sustainability: predicting the global distribution of Ralstonia solanacearum under current and future climate scenarios
Source: Front Plant Sci. 2025 Aug 4;16:1548640. doi: 10.3389/fpls.2025.1548640 (PMC12360038; doi:10.3389/fpls.2025.1548640)
Supplement: Supplementary file 1 [file DataSheet1.pdf]

Figures S1: Response curves of the most relevant environmental factors affecting the distribution of *Ralstonia solanacearum*; the shown values are average of ten replicate runs.

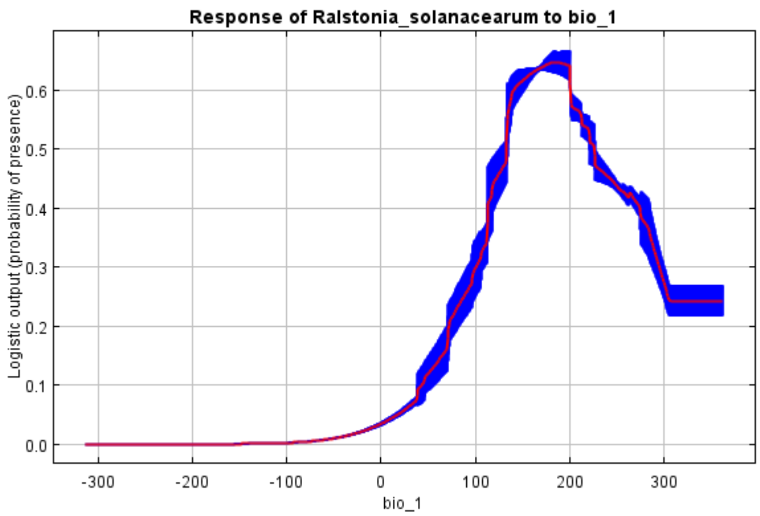

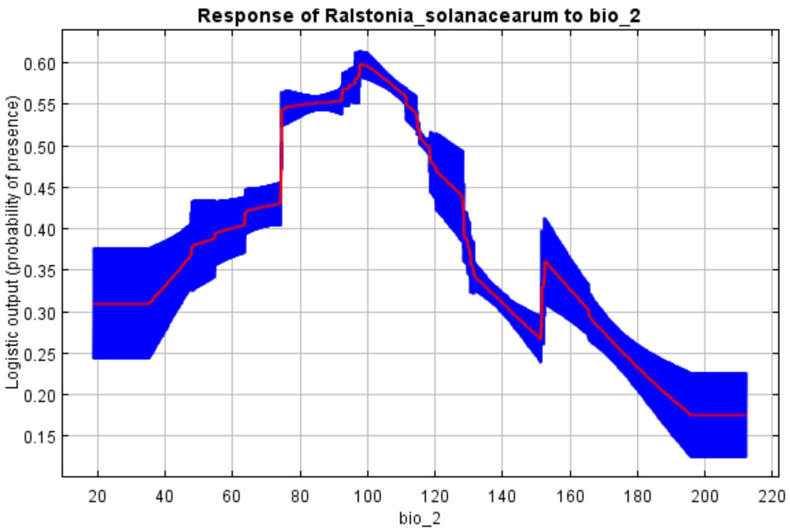

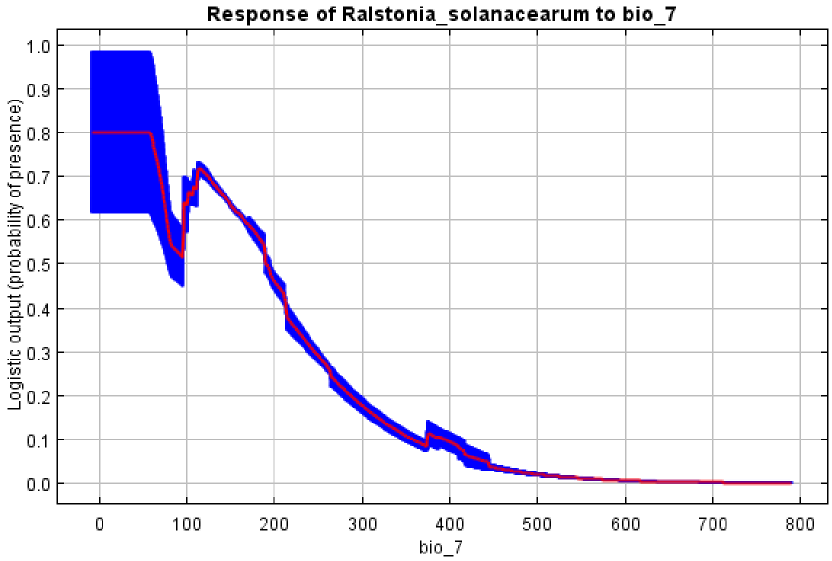
Response of Ralstonia_solanacearum to bio_7
Logistic output (probability of presence)
1.0
0.9
0.8
0.7
0.6
0.5
0.4
0.3
0.2
0.1
0.0
0
100
200
300
400
500
600
700
800
bio_7

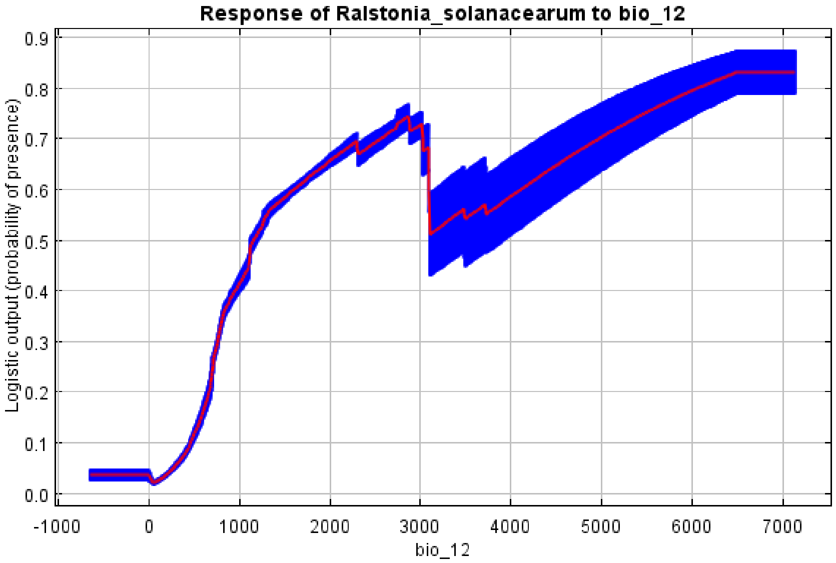
Response of Ralstonia_solanacearum to bio_12
Logistic output (probability of presence)
0.9
0.8
0.7
0.6
0.5
0.4
0.3
0.2
0.1
0.0
-1000
0
1000
2000
3000
4000
5000
6000
7000
bio_12

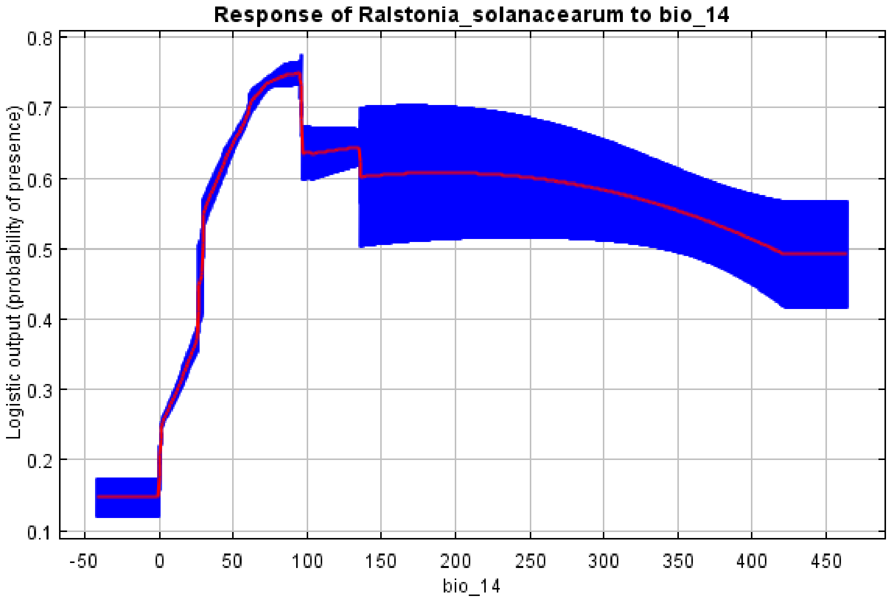

Response of Ralstonia_solanacearum to bio_14
Logistic output (probability of presence)
0.8
0.7
0.6
0.5
0.4
0.3
0.2
0.1
-50
0
50
100
150
200
250
300
350
400
450
bio_14
